# An unusual cause of renal failure; Epstein syndrome

**Published:** 2015-11-14

**Authors:** Manish R Balwani, Divyesh P Engineer, Manoj R Gumber, Vivek B Kute, Rajesh Singh, Himanshu V Patel, Aruna V Vanikar, Dinesh Gera, Pankaj R Shah, Hargovind L Trivedi

**Affiliations:** ^1^Department of Nephrology and Clinical Transplantation, Institute of Kidney Diseases and Research Center; ^2^Department of Pathology, Laboratory Medicine, Transfusion Services and Immunohematology, Dr. HL Trivedi Institute of Transplantation Sciences Ahmedabad, India

**Keywords:** Epstein Syndrome, Chronic kidney disease, Megathrombocytopenia, Hemodialysis, Deafness

## Abstract

Epstein syndrome constitutes macrothrombocytopenia without neutrophil inclusion bodies along with deafness and renal failure. A diagnosis of Epstein syndrome was made in a 17 year-old-male patient with macrothrombopathic thrombocytopenia, renal failure and sensorineural hearing loss. Our patient is unique as he presented with rapidly progressive renal failure and developed chronic kidney disease in second decade of life with no symptomatic hearing loss or bleeding tendency. Epstein syndrome needs to be differentiated from Alport syndrome which is more common disease with similar clinical presentation.

**Implication for health policy/practice/research/medical education:**

Thrombocytopenia in Epstein syndrome is not an absolute contraindication for major surgical procedures like arteriovenous fistula formation. Patients presenting with renal failure with thrombocytopenia should be screened for Epstein syndrome.

## Introduction

In 1972, Epstein et al (1) evaluated four patients who had hereditary nephritis and nerve deafness similar to Alport syndrome in association with macrothrombocytopenia. In this report we describe the presentation and clinical course of a young male with this rare disorder.

## Case presentation

**Figure 1:**
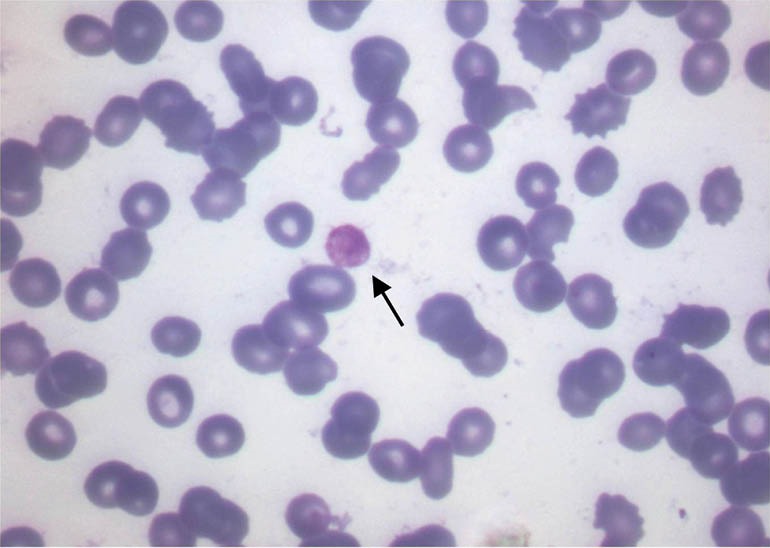
Peripheral smear showing megakaryocyte (arrow).

A 17-year-male presented with history of nausea, vomiting, shortness of breath, weakness and pedal oedema since 3 months. There was no history of decreased urine output, red urine, flank pain, skin rash, joint pain, nonsteroidal anti-inflammatory drug abuse or convulsion. There was no significant past medical history. Patient’s father died of renal failure at 40 years of age, cause of which was not known. Patient was pale and had pedal edema on admission. Blood pressure was 170/100 mm Hg. Rest of systemic examination was normal. Ophthalmic examination including fundus was normal, there was no evidence of lenticonus. Audiometry was suggestive of bilateral moderate to severe sensorineural hearing loss. Laboratory parameters revealed hemoglobin; 5.8 g/dl, total leucocyte count; 8130/µl, platelet count; 55 000/µl, blood urea; 61mg/dl, serum creatinine; 5.7 mg/dl, serum sodium; 134 meq/l, serum potassium; 5.1 meq/l, serum calcium; 8.1 mg/dl and serum phosphorous: 2.6 mg/dl. Urine analysis showed +3 albumin, urine pus cells; 5-6/hpf, urine red blood cell; 4-5/hpf with few granular casts. 24-hour proteinuria was 8.2 g/day. Liver function tests were normal. Serum parathyroid hormone level was 898 pg/dl. Serum ferritin level was 600 ng/dl. Peripheral smear for malarial parasite and malarial antigen detection test were negative. Immunological markers (ANA, anti-ds DNA, P-ANCA and C-ANCA) were negative. Complement level C3 and C4, serum lactate dehydrogenase were normal. Enzyme linked immunosorbent assay for hepatitis B, hepatitis C and human immunodeficiency virus were negative. Peripheral smear was negative for hemolysis and demonstrated giant platelets with thrombocytopenia (Figure 1). Coombs direct and indirect test were negative. Bone marrow examination was unremarkable. Bleeding time and clotting time was normal. Ultrasonography showed right and left side kidney size as 8.9 × 3.8 and 9.2 × 4.1 cm respectively with preserved corticomedullary differentiation. Doppler study of renal vessels was normal.

Double lumen catheter was inserted into right internal jugular vein at platelet count of 70 000/µl and patient was put on maintenance strict saline hemodialysis 2 to 3 times per week. Later arteriovenous fistula was created in left forearm at platelet count of 76 000/µl and there was no abnormal bleeding noted during these processes. Erythropoietin and intravenous iron sucrose supplement was given regularly. Patient’s blood pressure was maintained at 130/80 mm Hg with four anti-hypertensive drugs. He was explained about need for renal transplant.

## Discussion

Epstein et al described in 1972 patients with macrothrombocytopenia, nephritis and sensorineural deafness. He described two unrelated families with macrothrombocytopenia and mild hearing loss with renal disease similar to that of Alport syndrome (1). Autosomal dominant disorders with macrothrombocytopenias include May-Hegglin anomaly, Sebastian, Fechtner and Epstein syndrome (2). Epstein syndrome constitutes macrothrombocytopenia without neutrophil inclusion bodies along with deafness and renal failure (3). These platelet disorders are associated with mutations in the non-muscle myosin heavy chain 9 gene (MYH9) which encodes non-muscle myosin heavy chain IIA (MYHIIA) (2). The giant platelets in Epstein syndrome contain granules of normal structure with an irregular cytoplasm (4). Platelet survival is normal however aggregation and secretion in response to collagen, adenosine and thrombin are all decreased (4). Platelet function may vary from normal to grossly impaired (5). Patients may present with mild bleeding which can be easily corrected with platelet transfusion. Patients with Fechtner syndrome have similar clinical features but they show leucocyte inclusion bodies and may have cataract. Patients with Epstein syndrome usually present with renal abnormalities including microscopic hematuria, proteinuria, chronic kidney disease, hypertension or bleeding tendency. Progression to end stage renal failure between the third and fifth decade frequently ensues. Hearing abnormalities are usually uncovered during routine screening of patients (6,7).

Our patient presented with proteinuria, microscopic hematuria and granular casts in urine, he was diagnosed as chronic kidney disease and started on hemodialysis. Peripheral smear showed megathrombocytopenia. Hearing loss was detected on audiometry in the absence of such complaint by patient. In literature, renal pathological findings have been reported infrequently as the disease is accompanied by thrombocytopenia which makes renal biopsy unfeasible. Focal sclerosis glomerulonephritis is mostly reported among these patients (6). Renin-angiotensin system blockade is effective in reducing proteinuria of these patients (8). Successful cadaveric renal transplantation was carried out in a patient with Epstein syndrome by Alving et al (9). Hence, thrombocytopenia is not a contraindication for renal transplant. In our patient, arteriovenous fistula was created with no bleeding complication at platelet counts of 76  000/µl. There is no increased risk of thrombosis in such patients (10). Platelet transfusion, desmopressin and anti-fibrinolytic drugs can be used to decrease the risk of bleeding before surgery or invasive procedures. In diagnosed cases, urine analysis (including 24 hours protein) and measurement of serum creatinine prior to onset of renal disease on a yearly basis, audiometric and ophthalmologic evaluations every three years prior to onset of hearing loss and cataract, respectively is advised. Our patient is on maintenance hemodialysis thrice a week. Renal replacement therapy with both hemodialysis and peritoneal dialysis is a feasible option, along with renal transplantation. Prenatal diagnosis for a pregnancy at increased risk is possible if the disease-causing mutation in the family is known (11). Our patient is unique as he presented with rapidly progressive renal failure and developed chronic kidney disease in second decade of life with no symptomatic hearing loss or bleeding tendency. We have enrolled our patient for cadaver renal transplant as no living related donor is available.

## Conclusion

Patients presenting with renal failure and thrombocytopenia should be screened for Epstein syndrome. Thrombocytopenia in Epstein syndrome is not an absolute contraindication for major surgical procedures like arteriovenous fistula formation.

## Authors’ contribution

All authors wrote the paper equally.

## Conflicts of interest

The authors declared no competing interests.

## Ethical considerations

Ethical issues (including plagiarism, data fabrication, double publication) have been completely observed by the authors.

## Funding/Support

None.
